# Genome Sequence of a *Xylella fastidiosa* Strain Causing Mulberry Leaf Scorch Disease in Maryland

**DOI:** 10.1128/genomeA.00916-13

**Published:** 2014-03-06

**Authors:** Wei Guan, Jonathan Shao, Tingchang Zhao, Qi Huang

**Affiliations:** a Institute of Plant Protection, Chinese Academy of Agricultural Sciences, Beijing, China; b Floral and Nursery Plants Research Unit, U.S. Department of Agriculture, Agricultural Research Service, Beltsville, Maryland, USA; c Molecular Plant Pathology Laboratory, U.S. Department of Agriculture, Agricultural Research Service, Beltsville, Maryland, USA

## Abstract

*Xylella fastidiosa* causes bacterial leaf scorch in landscape trees, including mulberry. We determined the draft genome of the mulberry strain Mul-MD in order to gain a better understanding of the molecular basis of strain divergence, host specificity, nutrient requirements, and pathogenicity, as well as to develop genome-based specific detection methods.

## GENOME ANNOUNCEMENT

*Xylella fastidiosa* is a Gram-negative, nutritionally fastidious, insect-transmitted, and xylem-inhabiting bacterium that causes a wide range of plant diseases, including Pierce’s disease of grapevine and bacterial leaf scorch, in landscape trees such as mulberry. So far, eight *X. fastidiosa* genomes are available, including five complete genomes for the citrus variegated chlorosis strain 9a5c (1), Pierce’s disease strains Temecula 1 (2) and GB514 (3), and almond strains M12 and M23 (4), as well as three draft genomes for the oleander strain Ann1, the almond strain Dixon (5), and the *X. fastidiosa* biocontrol strain EB92-1 from elderberry (6). However, no genomes from landscape trees had been reported. We therefore sequenced the mulberry strain of *X. fastidiosa*, Mul-MD, which was isolated in 2011 from a mulberry tree displaying leaf scorch symptoms in Beltsville, MD.

Genomic DNA of *X. fastidiosa* strain Mul-MD was extracted from a triply cloned pure culture in periwinkle wilt medium (7) using a Blood and Tissue kit (Qiagen, Inc., Valencia, CA) according to the manufacturer’s instructions. Random shotgun and 3-kb mate-pair libraries of Mul-MD were generated and sequenced using Roche 454 GS (FLX titanium) pyrosequencing, resulting in 137,284 shotgun reads and 426,457 mate-pair reads totaling 254,742,009 bases, with a read-length average of about 450 bases. The total number of reads after processing by the Newbler Assembler from all libraries was 852,805 aligned reads, with 133,080,992 bases aligned. Using Newbler gsAssembler v 2.6, we assembled the genome into 188 contigs, of which 101 were >500 bases in size, and 27 scaffolds. The largest contig was 395,385 bases. Among the large contigs, the *N*_50_ contig size was 134,146 bases. The contigs have an average length of 13,528 bases and were run through the annotation pipeline, which uses GeneMark to predict coding regions based on prior *Xylella fastidiosa* gene models and runs BLASTX against a protein set that includes UniProt and all known *Xylella* proteins to determine edges of genes. The translated protein sequences were processed using Interproscan v. 4.8 for functional annotation and UniProt for additional descriptive information. Open reading frames shorter than 150 bases were eliminated. Selected open reading frames from *in silico* analysis that were not consistent with annotated *Xylella* genes were manually annotated. tRNA and rRNA predictions were made using the latest tRNAscan-SE and RNAmmer servers, respectively.

The 5× draft genome of the *X. fastidiosa* strain Mul-MD contains 2,543,372 bp and has a GC content of 51.65%. A total of 2,286 protein-encoding genes are predicted, 1,437 of which have tentatively been assigned a function. In addition, an ~25-kb plasmid sequence was found that is similar to the four plasmids, pXF-RIV11, pXF-RIV16, pXF-RIV19, and pXF-RIV25, present individually in four California mulberry strains of *X. fastidiosa* (8), as well as to the plasmid associated with the grapevine GB514 strain of *X. fastidiosa*.

### Nucleotide sequence accession numbers.

This whole-genome shotgun sequence has been deposited at DDBJ/EMBL/GenBank under the accession number AXDP00000000. The version described in this paper is version AXDP01000000.
